# Synaptic Delays for Insect-Inspired Temporal Feature Detection in Dynamic Neuromorphic Processors

**DOI:** 10.3389/fnins.2020.00150

**Published:** 2020-02-28

**Authors:** Fredrik Sandin, Mattias Nilsson

**Affiliations:** Embedded Intelligent Systems Lab (EISLAB), Luleå University of Technology, Luleå, Sweden

**Keywords:** pattern recognition, spiking neural network (SNN), neuromorphic, delay line, embedded intelligence, DYNAP, insect-inspired computing

## Abstract

Spiking neural networks are well-suited for spatiotemporal feature detection and learning, and naturally involve dynamic delay mechanisms in the synapses, dendrites, and axons. Dedicated delay neurons and axonal delay circuits have been considered when implementing such pattern recognition networks in dynamic neuromorphic processors. Inspired by an auditory feature detection circuit in crickets, featuring a delayed excitation by post-inhibitory rebound, we investigate disynaptic delay elements formed by inhibitory–excitatory pairs of dynamic synapses. We configured such disynaptic delay elements in the DYNAP-SE neuromorphic processor and characterized the distribution of delayed excitations resulting from device mismatch. Interestingly, we found that the disynaptic delay elements can be configured such that the timing and magnitude of the delayed excitation depend mainly on the efficacy of the inhibitory and excitatory synapses, respectively, and that a neuron with multiple delay elements can be tuned to respond selectively to a specific pattern. Furthermore, we present a network with one disynaptic delay element that mimics the auditory feature detection circuit of crickets, and we demonstrate how varying synaptic weights, input noise and processor temperature affect the circuit. Dynamic delay elements of this kind open up for synapse level temporal feature tuning with configurable delays of up to 100 ms.

## 1. Introduction

Processing of temporal patterns in signals is a central task in perception, learning, and control of behavior in both biological and technological systems (Indiveri and Sandamirskaya, 2019). Unlike digital circuits, which are designed to perform precise logic and arithmetic operations, neurons are unreliable, stochastic and slow information processing entities which form networks that function reliably through distributed information processing and adaptation. Neural circuits are therefore interesting models for development of mixed signal analog–digital processing and perception systems implemented in resource efficient nano-electronic substrates that are subject to device mismatch and failure (Strukov et al., 2019). In particular, energy-efficient neuromorphic processors and sensor systems have been developed by matching the device dynamics to neural dynamics, for example in the form of CMOS analog circuits operating in the subthreshold regime where semiconductor electron diffusion mimics ion diffusion in biological ion channels (Mead, 1990; Indiveri et al., 2011; Schuman et al., 2017). The dynamic nature and spatial structure of biological neurons (synapses, dendrites, axons, etc.) implies that neurons are inherently capable of temporal pattern recognition (Mauk and Buonomano, 2004) and pattern generation, also without recurrent connections. Furthermore, the event-driven neurons in Spiking Neural Networks (SNNs) are typically sparsely activated and offer an efficient way of doing inference (Rueckauer et al., 2017). SNNs with biologically plausible dynamics thus offer an interesting alternative model for temporal and spatial (spatiotemporal) pattern recognition (Pfeiffer and Pfeil, 2018), which can be further developed with guidance from biology. However, it is an open problem how such neuromorphic pattern recognition solutions can be engineered in practical applications such that the dynamic nature of the hardware is efficiently exploited.

Delays are essential for neuromorphic processing of temporal patterns in spike trains (Sheik et al., 2013) and have been studied since the early 90s, see for example the work by Van der Spiegel et al. (1994). Temporal delays have for example been implemented in neuromorphic processors in the form of multicompartment models (Hussain et al., 2015; Schemmel et al., 2017) and dedicated, specifically tuned delay neurons in the network architecture (Sheik et al., 2012a,b; Coath et al., 2014). In the latter case the resulting SNN is similar to a model of the auditory thalamocortical system described by Coath et al. (2011). Nielsen et al. (2017) present a low-power pulse delay and extension circuit for neuromorphic processors, which implements programmable axonal delays ranging from fractions of microseconds up to tens of milliseconds. In polychronous (Izhikevich, 2006) architectures, asynchronously firing neurons project to a common target along delay lines so that spikes arrive at the target neuron simultaneously, thus causing it to fire. A polychronous SNN with delay adaptation for spatiotemporal pattern recognition has been implemented in a Field-Programmable Gate Array (FPGA) and in a custom mixed-signal neuromorphic processor (Wang et al., 2013, 2014).

The typical signal propagation delays in axons (Swadlow, 1985) and dendrites (Agmon-Snir and Segev, 1993) of cortical neurons range up to tens of milliseconds. Furthermore, the dynamics of synapses also play an important role for the processing of temporal and spatiotemporal patterns (Mauk and Buonomano, 2004) and offer efficient dynamic mechanisms for sequence detection and learning (Buonomano, 2000). Synaptic dynamics enable pattern recognition architectures with high fan-in, which is beneficial in neuromorphic systems where multicompartment modeling, axon/neuron reservation and spike transmission is costly. Rost et al. (2013) present an SNN architecture with spike frequency adaptation and synaptic short-term plasticity that models auditory pattern recognition in cricket phonotaxis. There, synaptic short-term depression and potentiation is implemented to make neurons act as high-pass and low-pass filters, respectively. The resulting signals are combined in a neuron that acts as a band-pass filter and thereby responds to a frequency band that is matched to the particular sound pulse period of the crickets. Insects offer interesting opportunities to develop neuromorphic systems by modeling and finding guidance from their neural circuits, where the relatively low complexity allows neuromorphic engineers to transfer the principles of neural computation to applications (Dalgaty et al., 2018).

Our present investigation is inspired by a more recent description of the cricket auditory system (Schöneich et al., 2015) and preliminary work (Nilsson, 2018) indicating that dynamic synapses in a neuromorphic processor can be used to imitate the post-inhibitory rebound of the non-spiking delay neuron in the auditory circuit of the cricket. We configured disynaptic delay elements composed of inhibitory and excitatory dynamic synapses in the low-power Dynamic Neuromorphic Asynchronous Processor (DYNAP) model SE from aiCTX (Moradi et al., 2018). DYNAP-SE features reconfigurable mixed-mode analog/digital neuron and synapse circuits with biologically faithful dynamics. We investigated the properties and parameter dependence of the disynaptic delay elements in a population of neuromorphic neurons and found that delayed excitations of up to 100 ms can be achieved, and that the parameters can be selected so that the delay and delayed excitation amplitude depends mainly on the synaptic efficacies. Furthermore, we imitated the post-inhibitory rebound of the non-spiking neuron in the auditory circuit of the cricket (Schöneich et al., 2015) with one disynaptic element and investigated a circuit with three spiking neurons that reliably detects the species-specific sound-pulse interval of 20 ms. Since delays of tens of milliseconds are useful for implementing different kinds of neural circuits, cortical circuits in particular, the easily configurable properties of the disynaptic delay elements described and characterized in the following open up for further implementations and studies of SNN architectures in neuromorphic processors.

## 2. Materials and Methods

### 2.1. The DYNAP-SE Neuromorphic Processor

The DYNAP-SE neuromorphic processor uses a combination of low-power, inhomogeneous sub-threshold analog circuits and fast, programmable digital circuits for the emulation of SNN architectures with bio-physically realistic neuronal and synaptic behaviors (Moradi et al., 2018), making it a platform for spike-based neural processing with co-localized memory and computation (Indiveri and Liu, 2015). Specifically, the DYNAP-SE comprises four-core neuromorphic chips, each with 1k analog silicon neuron circuits. Each neuron has a Content-Addressable Memory (CAM) block containing 64 addresses representing the presynaptic neurons that the neuron is connected to. Information about spike-activity is transmitted between neurons in an Address-Event Representation (AER) digital routing scheme. Four different types of synaptic behavior are available for each connection: Fast excitatory, slow excitatory, subtractive inhibitory, and shunting inhibitory. The dynamic behaviors of the neuronal and synaptic circuits of the DYNAP-SE are governed by analog circuit parameters which are set by programmable on-chip temperature compensated bias-generators (Delbruck et al., 2010).

The inhomogeneity of the analog low-power circuits that constitute the neurons and synapses of the DYNAP-SE neuromorphic processor is due to device mismatch, and gives rise to variations in the dynamic behaviors of the silicon neurons and synapses that the analog circuits constitute. These variations are analogous to differences in values of the parameters governing the differential equations that model the neuronal and synaptic dynamics implemented in the chips. Consequently, one set value of a neuronal or synaptic bias parameter, in one core of the DYNAP-SE, results in a distribution of the corresponding parameter values in the population of neurons and synapses of that core.

#### 2.1.1. Spiking Neuron Model

In the DYNAP-SE, neurons are implemented according to the Adaptive Exponential Integrate-and-Fire (AdEx) spiking neuron model (Brette and Gerstner, 2005). The model describes the neuron membrane potential, *V*, and the adaptation variable, *w*, with two coupled non-linear differential equations,

(1a) $$\begin{array}{l} C\frac{dV}{dt}=-g_{L}\left(V-E_{L}\right)+g_{L}\Delta_{T}e^{\left(V-V_{T}\right)/\Delta_{T}}-w+I, \end{array}$$

(1b) $$\begin{array}{l} \tau_{w}\frac{dw}{dt}=a\left(V-E_{L}\right)-w, \end{array}$$

where *C* is the membrane capacitance, *g*_*L*_ the leak conductance, *E*_*L*_ the leak reversal potential, *V*_*T*_ the spike threshold, Δ_*T*_ the slope factor, *I* the (postsynaptic) input current, τ_*w*_ the adaptation time constant, and *a* the subthreshold adaptation. The membrane potential increases rapidly for *V* > *V*_*T*_ due to the non-linear exponential term, which leads to rapid depolarization and spike generation at time *t* = *t*_*spike*_, where the membrane potential and adaptation variable are updated according to

(2a) $$\begin{array}{l} V\to V_{r}, \end{array}$$

(2b) $$\begin{array}{l} w\to w+b, \end{array}$$

where *V*_*r*_ is the reset potential and *b* is the spike-triggered adaptation.

#### 2.1.2. Dynamic Synapse Model

In the DYNAP-SE, synapses are implemented with sub-threshold Differential Pair Integrator (DPI) log-domain filters proposed by Bartolozzi and Indiveri (2007) and further described by Chicca et al. (2014). The response of the DPI for an input current *I*_*in*_ can be approximated with a first-order linear differential equation,

(3) $$\begin{array}{l} \tau \frac{d}{dt}I_{out}+I_{out}=\frac{I_{th}}{I_{\tau }}I_{in}, \end{array}$$

where *I*_*out*_ is the (postsynaptic) output current, τ and *I*_τ_ are time constant parameters, and *I*_*th*_ is an additional control parameter that can be used to change the gain of the filter. This approximation is valid in the domain where *I*_*in*_≫*I*_τ_ and *I*_*out*_≫*I*_*I*_*th*__. The AdEx neuron model and the synapse equation are used in the following to describe the disynaptic delay elements that we configure in the DYNAP-SE in order to approximate the cricket auditory feature detection circuit.

### 2.2. Cricket Auditory Feature Detection Circuit

**Figure 1:**
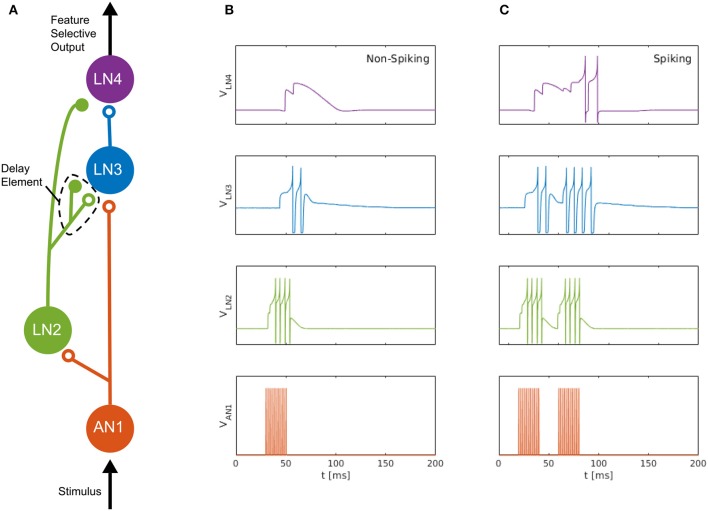
Neuromorphic feature detection circuit inspired by an auditory feature detection circuit in field crickets. **(A)** SNN architecture comprising four spiking neurons, on which open circles and solid disks denote, respectively, excitatory and inhibitory synapses. The disynaptic delay element imitates the dynamics of the non-spiking delay neuron, LN5, in the feature detection circuit of the cricket (Schöneich et al., 2015). **(B)** Measured neuron membrane potentials in the DYNAP-SE, following a 20-ms pulse stimulus. **(C)** Similarly, membrane potentials resulting from a pair of 20-ms stimulus pulses with a 20-ms interval, which causes the feature detecting neuron, LN4, to fire. By overcoming its inhibition and spiking, LN4 signals the feature detection.

We consider the auditory feature detection circuit for sound pattern recognition in the brain of female field crickets, described by Schöneich et al. (2015), which is used for the recognition of the sound pulse pattern of the male calling song. The circuit, consisting of five neurons, responds selectively to a species-specific sound-pulse interval of roughly 20 ms, by using a detection mechanism that relies on the coincidence of a direct neural response and a delayed response to the received sound pulses. In this circuit, a coincidence detecting neuron, LN3, receives excitatory projections along two separate pathways; one directly from the ascending neuron AN1, and the other via the inhibitory neuron LN2 followed by a non-spiking delay neuron LN5, which we approximate here with a delay element formed by an inhibitory–excitatory synapse pair, see Figure 1 (adapted from Nilsson 2018).

The non-spiking inhibitory neuron, LN5, in the cricket projects to LN3 and provides a delayed excitation of LN3 due to Postinhibitory Rebound (PIR). The duration of the delay matches that of the species-specific sound Interpulse Interval (IPI) of roughly 20 ms that the circuit is specialized for detecting, so that the delayed excitation arrives at the coincidence detecting neuron, LN3, simultaneously with the excitation caused by the subsequent sound pulse. The coincident excitations of LN3 enables it to fire and excite the feature detecting neuron, LN4, which, in turn, signals the feature detection by firing.

### 2.3. Disynaptic Delay Elements

The PIR of the non-spiking neuron LN5 in the cricket auditory feature detection circuit provides the delayed excitation of LN3 required for feature detection. For a general discussion of such delays, see Buonomano (2000) and Mauk and Buonomano (2004). Spike-based dynamic neuromorphic processors, such as the DYNAP-SE, cannot directly implement non-spiking neurons, such as the LN5 neuron in the cricket circuit, and flexible routing of such analog signals is problematic. Therefore, we approximate LN5 and PIR with an inhibitory–excitatory pair of dynamic synapses with different time constants, so that the sum of the two postsynaptic currents initially is inhibitory and subsequently becomes excitatory some time after presynaptic stimulation. For the inhibitory effect, a synapse of the subtractive type is used in the DYNAP-SE. As its name implies, the subtractive inhibitory synapse type allows for combining excitation and inhibition dynamics by summing inhibitory and excitatory postsynaptic currents, as opposed to the shunting synapse type which inhibits the neuron using a different mechanism. This summation of postsynaptic currents is the central mechanism of the proposed disynaptic delay element. For the excitatory part, the slow synapse type is used, leaving the fast synapse type available for bias configuration and use for stimulation of the postsynaptic neuron; in this case, for the projection from AN1 to LN3.

**Figure 2:**
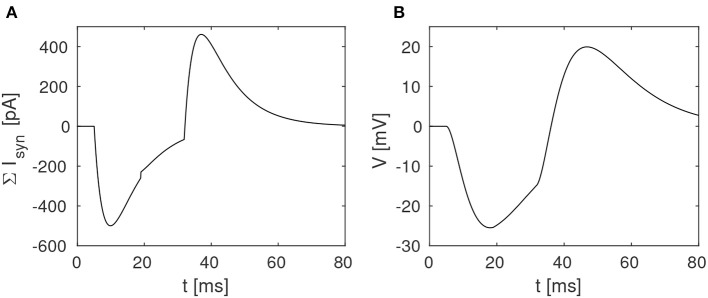
Simulation of the disynaptic delay element model. **(A)** Sum of inhibitory and excitatory postsynaptic currents from the delay element. **(B)** Resulting postsynaptic neuron membrane potential.

The proposed disynaptic delay element can be modeled with Equation (3), and the membrane potential resulting from presynaptic stimulation can be illustrated by solving Equation (1). Figure 2 shows a numerical simulation of the disynaptic delay element model for a 20 ms constant input current that represents the presynaptic stimulation, as in Figure 1.

Since the simulated neuron is in the subthreshold regime (*V* ≪ *V*_*T*_), Equation (1) is simplified by setting the exponential term to zero and omitting the adaption variable. The neuron and synapse parameters are selected so that the membrane potential is comparable to the potential measured in the hardware, and should thus not be directly compared with biological potentials and threshold values.

Dynamic disynaptic elements of this type are expected to provide a delayed excitation that qualitatively matches the effect of PIR in the output of non-spiking delay neurons like the LN5. Furthermore, we expect that the time delay and relative amplitude of inhibition and excitation can be configured, for example by modifying the synapse time constants and efficacies. The experimental results presented below demonstrate that this is indeed feasible, and that for some bias settings it is possible to control the time delay and delayed excitation amplitude with the synaptic efficacies only.

#### 2.3.1. Neuromorphic Implementation

The disynaptic delay elements were configured in the DYNAP-SE in two different ways. First, we aimed to mimic the post-inhibitory rebound in the cricket auditory circuit with a delay of about 20 ms. The delay elements were stimulated with four spikes equally spaced over the ~ 20-ms stimulus-response of LN2 for a 20-ms sound pulse, which represents the projection from LN2 to LN5 in the cricket circuit. The time constant of the inhibitory synapse of the delay element was set so that the resulting inhibition of LN3 corresponded to the inhibition caused by LN5 in the cricket; that is, a couple of ms longer than the 20-ms sound-pulse duration. The excitatory synapse was tuned so that the LN3 excitation lasts somewhat longer than that of the initial inhibition, approximately to the end of the corresponding PIR excitation of LN5 in the cricket. The weight of the inhibitory synapse was set higher than that of the excitatory synapse, such that the sum of inhibition and excitation turned out negative, thus inhibiting the neuron for the duration of the delay. For the excitatory synapse, the weight was set to yield a substantial excitation of the postsynaptic neuron following the inhibition, while not generating spikes without additional synaptic stimulation. In this manner, the effect of the non-spiking LN5 on LN3 is imitated with the summation of an inhibitory postsynaptic current and an excitatory postsynaptic current produced by two synapses on LN3. The resulting DYNAP-SE bias values are found in Table 1.

**Table 1:** Bias parameter values used for the characterization of individual disynaptic delay elements in the DYNAP-SE.

**Parameter type**	**Parameter name**	**Coarse value**	**Fine value**	**Current level**
Neuronal	IF_AHTAU_N	7	35	L
	IF_AHTHR_N	7	1	H
	IF_AHW_P	7	1	H
	IF_BUF_P	3	80	H
	IF_CASC_N	7	1	H
	IF_DC_P	0	40	H
	IF_NMDA_N	1	213	H
	IF_RFR_N	4	40	H
	IF_TAU1_N	5	39	L
	IF_TAU2_N	0	15	H
	IF_THR_N	6	4	H
Synaptic	NPDPIE_TAU_S_P	6	120	H
	NPDPIE_THR_S_P	1	30	H
	NPDPII_TAU_F_P	5	100	H
	NPDPII_THR_F_P	3	60	H
	PS_WEIGHT_EXC_S_N	1	110	H
	PS_WEIGHT_INH_F_N	1	130	H
	PULSE_PWLK_P	5	40	H
	R2R_P	4	85	H

Given the large parameter space of a dynamic neuromorphic processor like the DYNAP-SE, we then explored different ways to simplify the configuration of the disynaptic delay elements for delays up to about 100 ms. One identified possibility is to lower the constant injection current of the neurons receiving the delayed signal, to such an extent that the inhibition by the delay elements make the neuron reach its minimum membrane potential. This results in delay elements for which the duration of inhibition, τ_*inh*_, can be controlled with the inhibitory weight of the delay element, *w*_*inh*_. Furthermore, the amplitude of the post-inhibitory excitation, *V*_*max*_, is then controlled by the excitatory weight of the delay element, *w*_*exc*_, as well as by varying the number of presynaptic spikes stimulating the delay element. The DYNAP-SE bias values for this configuration of the delay elements are found in Table 2.

**Table 2:** Bias parameter values used for configuration of the disynaptic delay elements in the DYNAP-SE.

**Parameter type**	**Parameter name**	**Coarse value**	**Fine value**	**Current level**
Neuronal	IF_DC_P	1	30	H
Synaptic	NPDPIE_TAU_S_P	7	210	H
	NPDPIE_THR_S_P	1	30	H
	NPDPII_TAU_F_P	6	80	H
	NPDPII_THR_F_P	3	60	H
	PS_WEIGHT_EXC_S_N	0	8–80	H
	PS_WEIGHT_INH_F_N	0	1–150	H
	PULSE_PWLK_P	5	40	H
	R2R_P	4	85	H

*Neuronal parameters not defined in this table were set according to Table 1*.

#### 2.3.2. Characterization

**Figure 3:**
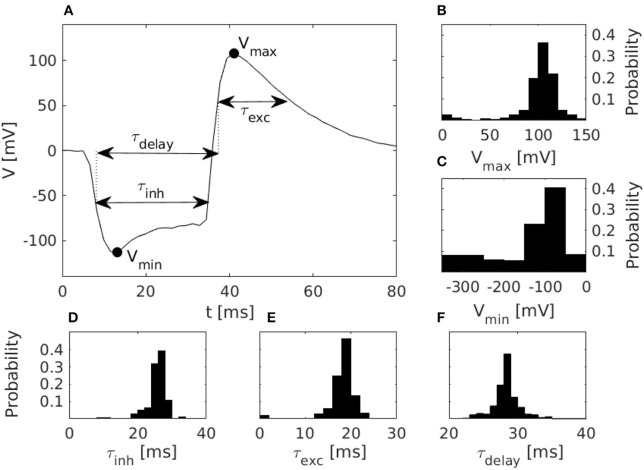
Characteristics of disynaptic delay elements configured in the DYNAP-SE neuromorphic processor. **(A)** Postsynaptic membrane potential vs. time, illustrating the delayed excitation resulting from a presynaptic pulse. **(B)** Distribution of the maximum measured membrane potential, *V*_*max*_, resulting from a presynaptic pulse. **(C)** Similarly, the distribution of the minimum measured membrane potential, *V*_*min*_. **(D)** Distribution of the inhibitory timescale, τ_*inh*_, defined as the full width at half minimum. **(E)** Distribution of the excitatory timescale, τ_*exc*_, defined as the full width at half maximum. **(F)** Distribution of the delay time, τ_*delay*_, defined as the duration from the onset of τ_*inh*_ to the offset of τ_*exc*_. The distributions in panels **(B–F)** were obtained via characterization of one DYNAP-SE core, comprising, in parallel, one disynaptic delay element on each of the 256 neurons, with biases configured according to Table 1.

For the purpose of characterization, the proposed disynaptic delay elements were implemented, in parallel, in one core of a DYNAP-SE neuromorphic processor; one delay element on each of the 256 neurons in the core. All of these neurons were then stimulated as described in section 2.3.1, and their membrane potentials were measured with an oscilloscope. To avoid oscilloscope and DYNAP-SE time synchronization issues, we analyzed the membrane potential measurements without reference to the precise timing of the presynaptic stimulation. The full duration at half minimum of the inhibition and the full duration at half maximum of the subsequent excitation, see Figure 2, can be determined from membrane potential measurements without reference to the timing of presynaptic spikes. Thus, we define the timescales of inhibition and delayed excitation in terms of the Full Duration at Half Maximum/Minimum (FDHM). We characterized the disynaptic delay elements with the distributions of the following five quantities: the minimum membrane potential, *V*_*min*_, the maximum membrane potential, *V*_*max*_, the FDHM of inhibition, τ_*inh*_, the FDHM of excitation, τ_*exc*_, and the time duration from the FDHM onset of the inhibition to the FDHM onset of the excitation, τ_*delay*_. These quantities are illustrated in Figure 3, and allowed us to investigate the effect of different bias parameter settings on the disynaptic delay elements in a population of neurons in the DYNAP-SE. This way the bias parameter values of the delay elements could for example be tuned to imitate the behavior of the delay neuron LN5 in the cricket. Further details on the experimental settings are described in section 2.5.

### 2.4. Neuromorphic Feature Detection Circuits

#### 2.4.1. Cricket Circuit

For the implementation of the cricket auditory feature detection circuit, as described in section 2.2, in the DYNAP-SE neuromorphic processor, stimuli representing the projections from AN1 upon auditory stimulation were generated in the form of 11 spikes evenly distributed over the pulse duration of 20 ms (in the noise-free case), yielding 10 Interspike Intervals (ISIs) of 2 ms each. Each of the remaining three neurons of the circuit, see Figure 1, were modeled on separate cores in one chip of the DYNAP-SE. The DYNAP-SE bias parameter values for the neurons LN2, LN3, and LN4 are found in Tables 3–5, respectively, and the neuromorphic implementations of these neurons are described in the following.

**Table 3:** Bias parameter values used for the inhibitory neuron, LN2, in the DYNAP-SE implementation of the cricket feature detection network.

**Parameter type**	**Parameter name**	**Coarse value**	**Fine value**	**Current level**
Neuronal	IF_AHTAU_N	7	35	L
	IF_AHTHR_N	7	1	H
	IF_AHW_P	7	1	H
	IF_BUF_P	3	80	H
	IF_CASC_N	7	1	H
	IF_DC_P	7	2	H
	IF_NMDA_N	7	1	H
	IF_RFR_N	4	208	H
	IF_TAU1_N	6	21	L
	IF_TAU2_N	5	15	H
	IF_THR_N	3	20	H
Synaptic	NPDPIE_TAU_F_P	5	165	H
	NPDPIE_THR_F_P	1	100	H
	PS_WEIGHT_EXC_F_N	0	190	H
	PULSE_PWLK_P	0	43	H
	R2R_P	4	85	H

**Table 4:** Bias parameter values used for the coincidence detecting neuron, LN3, in the DYNAP-SE implementation of the cricket feature detection network.

**Parameter type**	**Parameter name**	**Coarse value**	**Fine value**	**Current level**
Synaptic	NPDPIE_TAU_F_P	5	200	H
	NPDPIE_TAU_S_P	6	120	H
	NPDPIE_THR_F_P	1	30	H
	NPDPIE_THR_S_P	1	30	H
	NPDPII_TAU_F_P	5	100	H
	NPDPII_THR_F_P	3	60	H
	PS_WEIGHT_EXC_F_N	1	144–161	H
	PS_WEIGHT_EXC_S_N	1	110	H
	PS_WEIGHT_INH_F_N	1	130	H
	PULSE_PWLK_P	5	40	H
	R2R_P	4	85	H

*Neuronal parameters set according to Table 1*.

**Table 5:** Bias parameter values used for the feature detecting neuron, LN4, in the DYNAP-SE implementation of the cricket feature detection network.

**Parameter type**	**Parameter name**	**Coarse value**	**Fine value**	**Current level**
Synaptic	NPDPIE_TAU_F_P	5	80	H
	NPDPIE_THR_F_P	1	140	H
	NPDPII_TAU_F_P	6	180	H
	NPDPII_THR_F_P	3	140	H
	PS_WEIGHT_EXC_F_N	0	71–82	H
	PS_WEIGHT_INH_F_N	0	60	H
	PULSE_PWLK_P	0	43	H
	R2R_P	4	85	H

*Neuronal parameters set according to Table 3*.

For the implementation of the inhibitory neuron, LN2, a single neuron on a reserved core was used. This neuron was set to receive the generated stimulation representing AN1 by assigning a synaptic connection of the fast excitatory type. The bias parameter values from section 5.7.3 in the DYNAP-SE user guide ^1^ were used as reference. The parameter values of the fast excitatory synapse were then adjusted in order to model the behavior of LN2 as observed in the cricket. The synaptic time constant, NPDPIE_TAU_F_P, was adjusted to match that of the cricket, and the synaptic weight, PS_WEIGHT_EXC_F_N, and threshold parameter, NPDPIE_THR_F_P, were adjusted for LN2 to respond with the right amount of four to five spikes for each input pulse.

For the coincidence detecting neuron, LN3, the proposed delay elements were implemented according to the earlier description. An excitatory connection of the fast type was added for LN3 to receive the projection from AN1.

For the excitatory connection from LN3 to the feature detecting neuron LN4, a synapse of the fast type was used, and, for the inhibitory connection from LN2 to LN4, a synapse of the subtractive type was used. Bias parameter values from section 5.7.3 in the DYNAP-SE user guide were used for neuronal parameters, and as reference values for the fast excitatory synapses. For the fast inhibitory synapse, bias values from section 5.7.5 in the user guide were used as reference. The bias parameters, time constant, threshold and weight, for both synapse types, were then hand-tuned in order to approximate the behavior of LN4 in one DYNAP-SE neuron, so as to make LN4 spike, thus signaling feature detection, for stimuli with IPIs of 20 ms, but not for IPIs of 0, 10, 30, 40, and 50 ms.

#### 2.4.2. Single-Neuron Feature Detector

We further investigated the possibility that a single neuron in the DYNAP-SE with multiple disynaptic delay elements can respond selectively to spatiotemporal spike patterns, which match the difference in the delay times resulting from device mismatch. Specifically, we configured a neuron with two inputs via two different disynaptic delay elements. The input patterns consist of spike pairs, one spike for each delay element, with a variable spike-time interval. Patterns with spike-time intervals that match the delay-time difference between the two delay elements should generate postsynaptic currents with coincident maxima, thus resulting in maximum excitation of the neuron.

The neuron and delay elements were configured as described in section 2.3.1 with bias parameter values according to Table 2, with a few modifications: The threshold, IF_THR_N = (6, 135), and excitatory synaptic efficacy was modified so that the neuron generates output spikes for two input spikes, and the inhibitory weight of the delay elements was modified accordingly. Furthermore, the time-constant of the excitatory synapse was lowered to compensate for the strong excitation required, NPDPIE_TAU_S_P = (5, 70) and NPDPIE_THR_S_P = (0, 210). The numbers in parentheses denote coarse and fine parameter values of the DYNAP-SE, respectively.

The synapses were selected with an off-line Hebbian-like learning rule such that, for the spike patterns considered, the neuron responded selectively to spike patterns with intermediately long intervals, but not to spike patterns with shorter or longer intervals. Spike patterns were generated as described in the next section, and the neuron was stimulated one hundred times with each pattern. Based on these experiments the average probability of the neuron to spike for each type of pattern was determined.

### 2.5. Experiments

In all of the experiments conducted in this work, the DYNAP-SE neuromorphic processor was controlled using the cAER event-based processing framework for neuromorphic devices. More specifically, a custom module making use of the tools for configuration and monitoring provided by cAER was created and added to the framework. All stimuli were synthetically generated using the built-in spike generator in the FPGA of the DYNAP-SE, which generates spike-events according to assigned ISIs and virtual source-neuron addresses.

The DYNAP-SE features analog ports for monitoring of neuron membrane potentials. For measurements of these potentials, the 8-bit USB oscilloscope SmartScope by LabNation was used. Since these measurements only capture the neuron membrane potential, there is no information about the precise relative timing of spike-events in the resulting data. Because of this, the durations of inhibition and excitation of the delay elements were defined in terms of the FDHM as described above.

For the extraction of the delay parameters defined in section 2.3.1, the stimulus was repeatedly broadcast to all neurons in the core, and for each stimulation cycle one neuron was monitored with the oscilloscope using the programmable analog outputs of the DYNAP-SE. The stimulation cycle was given a duration of 0.5 s, in order for the neurons to relax to a resting state before and after stimulation. At the initial state of rest, the resting potential was automatically estimated for each neuron. The resting potential was subsequently subtracted from the measurement data, such that the resulting resting potentials are zero. This was done to make the parameter values of the different neurons comparable with each other.

## 3. Results

### 3.1. Characteristics of Delay Elements

Results from the characterization of the disynaptic delay elements, implemented in parallel on each of the 256 neurons in one core of the DYNAP-SE neuromorphic processor, are presented in Figure 3.

The figure shows the pulse-response of one typical delay element from the resulting population, along with histograms of the distributions of parameters that characterize each delay element. The resulting values of *V*_*max*_ range from 3 to 143 mV and center around 105 mV. *V*_*min*_ has a thicker tail of the distribution and range from −310 to −20 mV, with most values between −100 and −50 mV. The time constant distributions have relatively thin tails. τ_*inh*_ has values between 6 and 47 ms with probability peaking between 26 and 28 ms. τ_*exc*_ ranges from 0 to 38 ms with probability peaking between 18 and 20 ms, and τ_*delay*_ spans between 22 and 51 ms with probability peaking between 28 and 29 ms.

**Figure 4:**
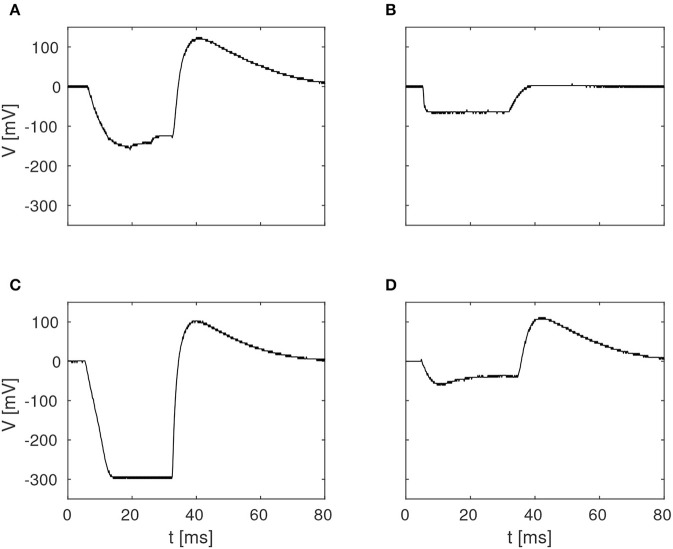
Examples of four different membrane potentials measured in the characterization of the delay elements summarized in Figure 3. These variations were observed in one core with 256 neurons, with biases configured according to Table 1.

The pulse-responses of four different delay elements are presented in Figure 4, which illustrates the variety of delay dynamics obtained thanks to device mismatch. Here, the variance of the minimum voltage, *V*_*min*_, is especially evident, but variation in other parameters can also be observed, such as *V*_*max*_, in the case of the virtually non-existing excitation in Figure 4B.

### 3.2. Cricket Feature Detection

**Figure 5:**
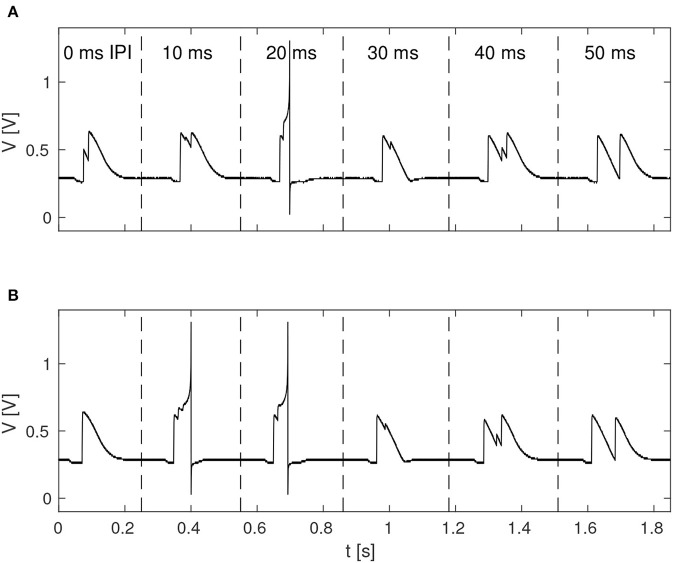
Response of LN4 for double-pulse stimuli with IPIs of 0, 10, 20, 30, 40, and 50 ms, respectively. **(A)** Noiseless case. **(B)** Example for 20% noise, with a false positive for the 10-ms IPI.

The function of the neuromorphic implementation of the feature detection SNN was investigated by stimulating it with double pulses of 20 ms duration each, while increasing the IPI from 0, 10, 20, 30, 40, to 50 ms. Furthermore, in order to investigate the effect of noise in the stimuli, as is likely to be present in real-world environments, different levels of spike-timing noise was introduced in the generated stimuli by randomly perturbing the value of the ISIs with values drawn from a continuous uniform distribution. Figure 5 shows the membrane potential of LN4 during correct classification of noiseless double pulses of all of the IPIs mentioned above, as well as the result in the presence of 20% spike-timing noise, where some false positives are observed for the 10 ms IPI.

**Figure 6:**
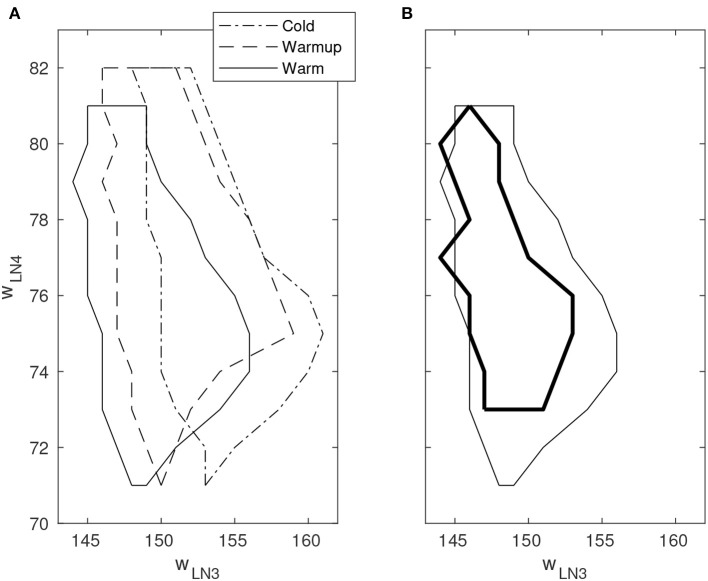
Boundary of correct stimulus classification in synaptic parameter space. Outside the enclosed region, false positives and/or false negatives occur with varying probability. The horizontal and vertical axes indicate the fine integer bias-values of the excitatory synaptic weight for the neurons LN3 and LN4, respectively. Multiple line types indicate experiments performed under different environmental conditions. **(A)** Movement of the classification boundary observed after several hours of continuous operation from cold startup. The temperature change is likely caused by the FPGA that is enclosed in the system. **(B)** Shrinkage of the classification boundary in presence of 10% spike-timing noise in the stimulus (bold line). Boundary points are temperature dependent.

By varying the weights of the excitatory projection from AN1 to LN3 and the excitatory synaptic weight of LN4, respectively, a boundary of correct classification of stimuli could be identified in the space spanned by these two parameters. Outside the boundary, false positives and/or false negatives occur with varying probability. The boundary was observed to move substantially in the parameter space as time progressed after cold startup of the DYNAP-SE and this is likely due to heating by the FPGA that is enclosed in the DYNAP-SE system. This change was observed over multiple runs of the experiment and appears to be qualitatively consistent. Furthermore, the shift of the boundary in the presence of spike-timing noise in the stimuli was investigated. Figure 6 shows the boundary of correct classification, as measured at three separate points in time after device initialization, spanning from minutes to several hours of run-time. The figure also shows the shrinkage of the classification boundary in the presence of 10% spike-timing noise in the stimuli, in relation to the steady-state of the boundary after several hours of system run-time.

**Figure 7:**
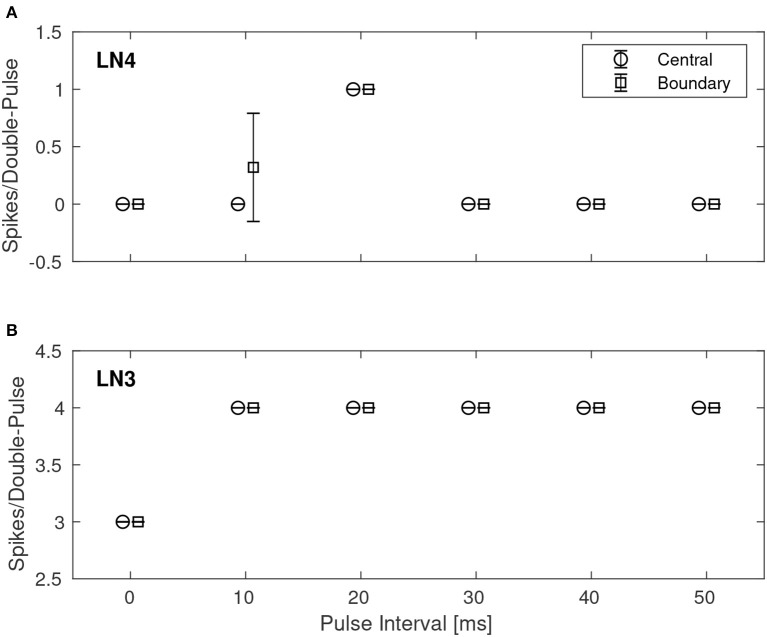
Average number of spikes from LN3 and LN4 per double-pulse stimulus for varying IPIs and two different bias configurations: one central to, and one on the boundary of, the region illustrated in Figure 6. For each IPI, the data-points are graphically separated by 4/3 ms to improve clarity of the visualization. Error bars denote ±1 standard deviation. **(A)** Feature detecting neuron, LN4. **(B)** Coincidence detecting neuron, LN3.

A quantitative investigation of the IPI dependence of the feature detection circuit was made by repeatedly stimulating the network with double pulses of different IPIs as described earlier, while observing the response in LN3 and LN4 by recording and counting the spikes of both neurons. For each IPI, the network was presented with the corresponding double-pulse stimulus 50 times. Figure 7 shows, in the case of noiseless stimuli, the average number of spikes from LN3 and LN4, respectively, centrally within the synaptic boundary of correct classification, as well as at the boundary. Centrally within the boundary of correct classification, LN4 responded exclusively to the 20 ms pulse interval, with no false positives or negatives. On the boundary of the parameter space, LN4 began to exhibit false positives for the 10 ms IPI, with 0.32 ± 0.47 spikes per double-pulse stimulus.

**Figure 8:**
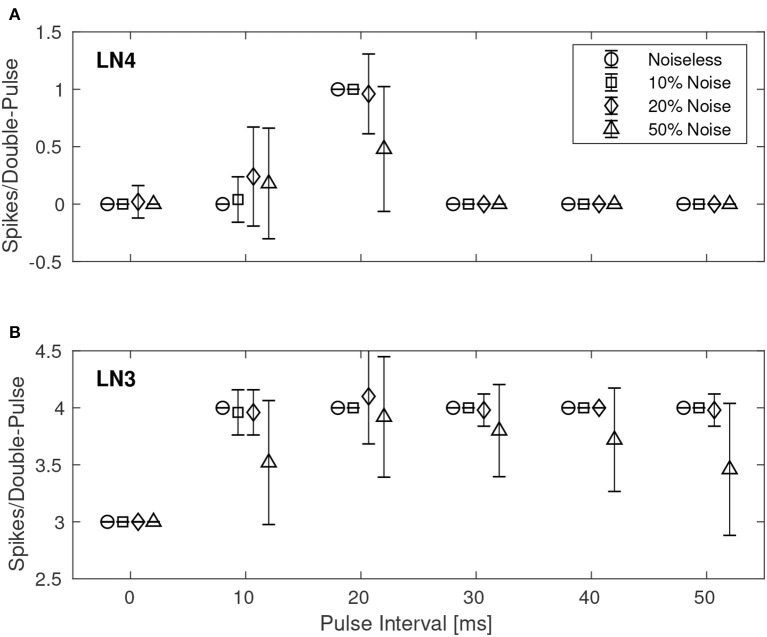
Average number of spikes from LN3 and LN4 per double-pulse stimulus for varying IPIs and different levels of spike-timing noise in the stimuli. For each IPI, the data-points are graphically separated by 4/3 ms to improve the clarity of the visualization. Error bars denote ±1 standard deviation. **(A)** Feature detecting neuron, LN4. **(B)** Coincidence detecting neuron, LN3.

Similarly, Figure 8 shows the results for the best synaptic configuration used in the previous experiment, centrally located within the boundary of correct classification, but for different levels of spike-timing noise. As expected the network performed correct classification in the noiseless case. The introduction of noise caused LN4 to exhibit false positives, in particular for the 10 ms IPI. At higher levels of noise also false negatives were observed. In the case of 50% noise the response of LN4 was 0.18 ± 0.48 spikes per double-pulse for the 10 ms IPI, and 0.48 ± 0.54 spikes for the 20 ms IPI.

### 3.3. Reconfigurability of Delay Elements

**Figure 9:**
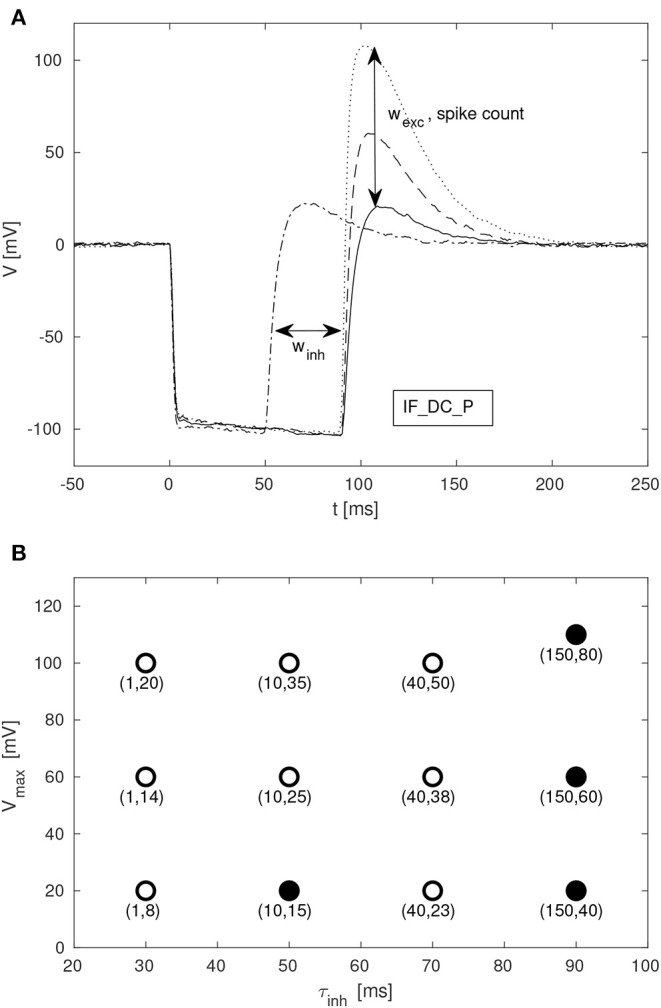
Configuration of disynaptic delay elements. **(A)** Postsynaptic membrane potential vs. time, resulting from a presynaptic pulse. The delay is controlled mainly by the inhibitory synaptic efficacy, *w*_*inh*_. The amplitude of the delayed excitation is controlled mainly by the excitatory synaptic efficacy, *w*_*exc*_, and by the number of presynaptic spikes. Note that the membrane potential reaches its minimum possible value during inhibition, and that the difference between this value and the resting potential is controlled with the constant injection current of the neuron, controlled by the bias parameter IF_DC_P. **(B)** Maximum membrane potential, *V*_*max*_, vs. duration of inhibition, τ_*inh*_, for different values of (*w*_*inh*_, *w*_*exc*_). Each point is denoted with the corresponding fine integer bias values of the inhibitory and excitatory synaptic weights, respectively.

Given the large parameter space of a dynamic neuromorphic processor, such as the DYNAP-SE, we explored different ways to simplify the configuration of the disynaptic delay elements for delays up to about 100 ms. Figure 9A shows four configurations of one delay element, with the maximum membrane potential of the post-inhibitory excitation ranging between 20 and 110 mV, and the durations of inhibition ranging between 50 and 90 ms, according to the FDHM definition.

A table with delay element weight values and resulting values of τ_*inh*_ and *V*_*max*_, from a total of 12 such variations, is presented in Figure 9B; the data-points corresponding to the membrane potentials in Figure 9A are marked with filled disks.

### 3.4. Feature Detection With Multiple Delay Elements

**Figure 10:**
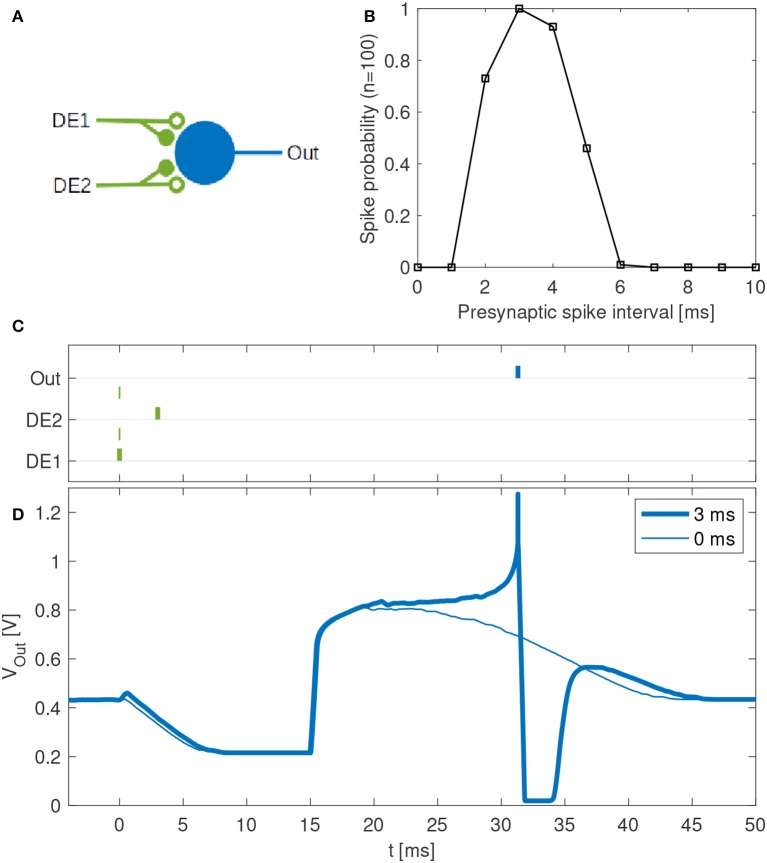
Feature detection by a single neuron in the DYNAP-SE. **(A)** Neuron with one output (Out) and two inputs with disynaptic delay elements (DE1 and DE2). **(B)** Probability that the neuron spikes vs. the presynaptic spike interval, which denotes the time between two presynaptic spikes at DE1 and DE2, respectively. This neuron spikes with maximum probability when a spike arrives to DE2 about 3 ms later than to DE1. The neuron does not spike for presynaptic spike intervals below about 2 ms and above about 6 ms. **(C)** Examples of spike times for presynaptic spike intervals of 3 ms (bold lines) and 0 ms (thin lines). In the latter case no postsynaptic spike is generated. **(D)** Examples of membrane potentials measured for 3 ms (bold line) and 0 ms (thin line) presynaptic spike intervals. No spike is generated when the two presynaptic spikes arrive simultaneously. With a presynaptic spike interval of 3 ms the neuron spikes reliably.

Disynaptic delay elements produce variable delayed excitations when stimulated with presynaptic spikes, as demonstrated in Figure 9. Furthermore, the delayed excitations are subject to device mismatch variability, as demonstrated in Figure 3. Thus, as described in section 2.4.2 we investigated the possibility that a single neuron with multiple disynaptic delay elements can respond selectively to spatiotemporal patterns that match the different delay times. We find that this is possible, and one example is illustrated in Figure 10, which shows the results for one neuron in DYNAP-SE with two delay elements (DE1 and DE2) stimulated with eleven different spatiotemporal patterns.

The experiment with each pattern is repeated one hundred times. The neuron fires selectively when the time interval between presynaptic spikes, *t*_*DE*2_ − *t*_*DE*1_, is 3 to 4 ms, while the probability of firing is low for shorter and longer presynaptic spike intervals. The neuron does not fire when *t*_*DE*2_ − *t*_*DE*1_ < 0.

## 4. Discussion

SNN architectures for temporal pattern recognition require delays, and the dynamics of synapses, dendrites and axons of cortical neurons correspond to a spectrum of signal propagation delays ranging up to about 100 ms. In this work, we investigate delays produced by inhibitory–excitatory pairs of conventional conductance-based dynamic synapses implemented in the DYNAP-SE neuromorphic processor. Our main results presented in Figures 3, 9, 10 demonstrates that configurable delayed excitations of up to about 100 ms can be implemented in this way, and that a single neuron with multiple disynaptic delay elements can respond selectively to spatiotemporal input patterns. Figure 3 illustrates that for one particular configuration of the disynaptic elements, which is selected to mimic the PIR of a particular non-spiking delay neuron in crickets, a distribution of delays are realized in one neuromorphic core thanks to device mismatch. Furthermore, Figure 9B illustrates a subset of the possible disynaptic configurations resulting in different delays (τ_*inh*_ = 30, 50, 70, 90 ms) and delayed excitation amplitudes. Thus, by configuring the two synaptic parameters of the disynaptic elements, variable excitation strengths and delays of up to about τ_*delay*_ ≃ 100 ms are achieved, which is similar to the range of dendritic and axonal signal propagation delays in cortical circuits (Dayan and Abbott, 2005).

At the quantitative level, we observe some differences between the feature detection results presented in section 3.2 and the behavior of the cricket circuit described by Schöneich et al. (2015). In the crickets, the response of the coincidence detector neuron LN3 for different IPIs varies so that the distribution of the number of spikes of LN3 increases as the interval gets closer to the species-specific IPI of 20 ms. This is not the case in the results presented here, and further optimization of the neuron and synapse parameters are required if this behavior is to be imitated. As illustrated in Figure 7B, our LN3 reliably produces the same number (but different timings) of spikes for all of the different IPIs, with the exception of the 0 ms IPI. A more plausible trend is observed in the case of 50% input noise, but in that case the classification results are weaker. Hence, the classification mechanism relies on the timing of spikes and the balance of inhibition and excitation.

Temporal feature detection and pattern recognition are central tasks in advanced sensor and perception systems. Thus, low-power SNN processors enabling learning and recognition of complex spatiotemporal patterns (Indiveri and Sandamirskaya, 2019; Strukov et al., 2019) have many potential applications, for example for always-on machine monitoring (Martin del Campo et al., 2013; Martin del Campo and Sandin, 2017), where the system needs to operate autonomously and wirelessly with limited resources over the expected lifetime of the monitored machine component (Martin del Campo, 2017; Häggström, 2018). Although we sidestep Dale's principle, the dynamic disynaptic delay elements investigated here have the desirable property that each neuron can be configured with multiple disynaptic elements, as illustrated in Figure 10. By combining multiple disynaptic delay elements, for example in line with the idea of polychronous networks (Izhikevich, 2006), more complex spatiotemporal patterns can be detected in principle. Since the disynaptic delay elements are realized with ordinary dynamic synapses, the approach is not limited to this particular neuromorphic processor, although the distribution of delays obtained is processor and device-mismatch dependent.

Further work is required to investigate how the repertoire of synaptic delays can be exploited and configured/learned to solve practical pattern recognition tasks, and to further develop the understanding of how device mismatch, noise and temperature variations affect different network architectures. With dynamic synapses featuring short- and long-term plasticity, additional mechanisms for sequence detection and learning can also be realized (Buonomano, 2000) and investigated. Furthermore, SNNs can faithfully reproduce dynamics of brain networks, which appear to self-organize near a critical point where no privileged spatial or temporal scale exist, which has interesting consequences for information processes (Cocchi et al., 2017). Thus, Neuromorphic Engineering (Indiveri and Horiuchi, 2011; Strukov et al., 2019) and dynamic neuromorphic processors opens the way to new interesting architectures for pattern recognition and generation in machine perception and control.

## Data Availability Statement

The raw data supporting the conclusions of this article will be made available by the authors, without undue reservation, to any qualified researcher.

## Author Contributions

FS conceived the possibility to imitate non-spiking PIR delay in the DYNAP-SE with synaptic dynamics, supervised the experiments to be carried out, and wrote part of the manuscript. MN implemented the code that controls the DYNAP-SE, performed the experiments, and wrote part of the manuscript.

### Conflict of Interest

The authors declare that the research was conducted in the absence of any commercial or financial relationships that could be construed as a potential conflict of interest. The reviewer FC declared providing technical help to the authors with the material they used in their research, with no collaboration, before the review.

## Footnotes

^1^https://aictx.ai/technology/
